# Comparative Immunogenicity of BNT162b2 mRNA Vaccine with Natural SARS-CoV-2 Infection

**DOI:** 10.3390/vaccines9091017

**Published:** 2021-09-13

**Authors:** Mina Psichogiou, Andreas Karabinis, Garyphallia Poulakou, Anastasia Antoniadou, Anastasia Kotanidou, Dimitrios Degiannis, Ioanna D. Pavlopoulou, Antigoni Chaidaroglou, Sotirios Roussos, Elpida Mastrogianni, Irene Eliadi, Dimitrios Basoulis, Konstantinos Petsios, Konstantinos Leontis, Eleni Kakalou, Konstantinos Protopapas, Edison Jahaj, Maria Pratikaki, Konstantinos N. Syrigos, Pagona Lagiou, Helen Gogas, Sotirios Tsiodras, Gkikas Magiorkinis, Dimitrios Paraskevis, Vana Sypsa, Angelos Hatzakis

**Affiliations:** 1 First Department of Internal Medicine, Laiko General Hospital, Medical School, National and Kapodistrian University of Athens, 11527 Athens, Greece; elpidoulama@hotmail.com (E.M.); eirini.iliadi@gmail.com (I.E.); dimitris.bassoulis@gmail.com (D.B.); helgogas@gmail.com (H.G.); 2 Onassis Cardiac Surgery Center, 17674 Athens, Greece; karabinis291@hotmail.com (A.K.); degiannis@yahoo.com (D.D.); achaidaroglou@yahoo.com (A.C.); 3 3rd Department of Internal Medicine, Sotiria General Hospital, Medical School, National and Kapodistrian University of Athens, 11527 Athens, Greece; gpoulakou@gmail.com (G.P.); kostas.leontis@hotmail.com (K.L.); ekakalou@gmail.com (E.K.); ksyrigos@med.uoa.gr (K.N.S.); 4 4th Department of Internal Medicine, Medical School, National and Kapodistrian University of Athens, 12462 Athens, Greece; ananto@med.uoa.gr (A.A.); kprotopapas@hotmail.com (K.P.); sotirios.tsiodras@gmail.com (S.T.); 5 1st Department of Critical Care & Pulmonary Services, Medical School, National and Kapodistrian University of Athens, Evangelismos Hospital, 10676 Athens, Greece; akotanid@gmail.com (A.K.); edison.jahaj@gmail.com (E.J.); mpratikaki@yahoo.com (M.P.); 6 Pediatric Research Laboratory, Faculty of Nursing, National and Kapodistrian University of Athens, 11527 Athens, Greece; idpavlop@yahoo.gr; 7 Department of Hygiene, Epidemiology and Medical Statistics, Medical School, National and Kapodistrian University of Athens, 11527 Athens, Greece; sotirisroussos@yahoo.co.uk (S.R.); pdlagiou@med.uoa.gr (P.L.); gmagi@med.uoa.gr (G.M.); dparask@med.uoa.gr (D.P.); vsipsa@med.uoa.gr (V.S.); ahatzak@med.uoa.gr (A.H.); 8 Clinical Research Office, Onassis Cardiac Surgery Center, 17674 Athens, Greece; petsiosk@gmail.com; 9 Hellenic Scientific Society for the Study of AIDS, Sexually Transmitted and Emerging Diseases, 11527 Athens, Greece

**Keywords:** COVID-19, BNT162b2 vaccine, health care workers, immune response, anti-RBD

## Abstract

BNT162b2 has proven to be highly effective, but there is a paucity of data regarding immunogenicity factors and comparison between response to vaccination and natural infection. This study included 871 vaccinated healthcare workers (HCW) and 181 patients with natural infection. Immunogenicity was assessed by measuring anti-SARS-CoV-2 against the RBD domain of the spike protein (anti-RBD). Samples were collected 1–2 weeks after vaccination or 15–59 days post-onset of symptoms. Post-vaccine anti-RBD concentrations were associated with age, gender, vaccination side-effects (VSE) and prior infection (Pr-CoV). Anti-RBD median levels (95%CI) were lower by 2466 (651–5583), 6228 (3254–9203) and 7651 (4479–10,823) AU/mL in 35–44, 45–54, 55–70 yrs, respectively, compared with the 18–34 yrs group. In females, the median levels were higher by 2823 (859–4787), 5024 (3122–6926) in individuals with VSE, and 9971 (5158–14,783) AU/mL in HCWs with Pr-CoV. The ratio of anti-RBD in vaccinated individuals versus those with natural infection varied from 1.0 to 19.4. The high immunogenicity of BNT162b2 is verified, although its sustainability has yet to be elucidated. The use of comparative data from natural infection serological panels, expressing the clinical heterogeneity of natural infection, may facilitate early decisions for candidate vaccines to be evaluated in clinical trials.

## 1. Introduction

The coronavirus disease 2019 (COVID-19) is expanding despite mitigation policies of variable levels of success. By the end of 2020, an increasing number of safe and effective vaccines were approved, and large vaccination programs are underway around the world. By 13 July 2021, more than 188,000,000 cases and 4,000,000 deaths were reported while more than 3.4 billion vaccine doses were administered.

The first approved vaccine was Pfizer-BNT162b2, a lipid-nanoparticle-formulated mRNA vaccine encoding the SARS-CoV-2 full length spike modified by two proline mutations. Preliminary findings among healthy men and women showed that two 30 mg doses elicited high SARS-CoV-2-neutralizing antibody titers and robust antigen-specific CD8+- and Th1-type CD4+ T-cell responses [1]. Moreover, in a multinational, placebo-controlled, observer-blinded efficacy trial including 43.548 healthy or stable-chronic-condition participants, the vaccine was found to be safe with an efficacy of 95% (95% credible interval 90.3–97.6%) in preventing symptomatic COVID-19 disease ≥7 days after the 2nd dose [2]. A similar vaccine efficacy (90–100%) was observed across groups defined by age, sex, race, baseline body mass index (BMI) and the presence of coexisting conditions. History of previous COVID-19 treatment, immunosuppressive therapy or diagnosis with an immunocompromising condition were exclusion criteria in this pivotal study.

A second approved mRNA vaccine, Moderna-mRNA 1273, showed similar efficacy 94.1% (95% 89.3–96.8) in preventing the COVID-19 illness [3].

The BNT162b2 was also evaluated in a mass vaccination campaign in Israel with an efficacy, 7 days from the 2nd dose, of 94%, 87% and 92% in preventing symptomatic disease, hospitalization, and severe disease, respectively [4].

The presence of neutralizing antibodies is a strong correlate of vaccine efficacy, although a protection threshold has not been established [5]. However, measuring neutralizing antibodies on a large scale is challenging. The development of binding assays directed against the spike protein of SARS-CoV-2 showed excellent correlation with neutralizing antibodies [6,7,8,9] and provides an opportunity to assess the immunogenicity of SARS-CoV-2 vaccines over time on a large scale. The assessment of vaccine immunogenicity to predict and monitor vaccine effectiveness is important in groups of individuals not included in clinical trials, such as patients with immunocompromising conditions [10].

Despite the high levels of vaccine efficacy in immunocompetent individuals, the duration of BNT162b2 protection remains unknown. Antibody titers in other coronaviruses (seasonal, SARS CoV-1, MERS) wane over time and this is the case with COVID-19 antibodies in natural infection [11,12,13].

Natural infection protects from reinfection for at least 7 months, and represents a benchmark for comparison with vaccine efficacy and immunogenicity [14].

Higher levels of neutralizing antibodies (NA) and binding antibodies have been associated with increased clinical severity of natural infection in several studies [14,15,16].

Phase I/II SARS-CoV-2 vaccine immunogenicity studies on approved or under approval vaccines included limited numbers of individuals with natural infection as a control group [1,17,18]. Moreover, the heterogeneity of natural infection was not considered and comparisons of vaccinated individuals with the former group were incomplete.

Herein, we report comparative immunogenicity data of BNT162b2 mRNA vaccine with a large cohort of individuals with natural COVID-19 infection and we provide suggestions for the use of comparative immunogenicity of candidate vaccines with natural infection for an early prediction of vaccine efficacy.

## 2. Materials and Methods

### 2.1. Vaccination for HCWs

Participants were vaccinated with 2 doses of BNT162b2 21 days apart. The vaccine was administered intramuscularly and included 30 mg of SARS-CoV-2 full-length lipid-nanoparticle-formulated mRNA.

The study was designed to assess immunogenicity at time intervals 1–2 weeks after the 2nd dose (28–35 days) and 4, 6, 8, and 12 months after the 1st dose. Immunogenicity 1–2 weeks after the 2nd dose was expected to be highest, based on the results from phase I/II studies [1].

HCWs from 2 teaching hospitals, Laiko General Hospital (Hospital 1) and Onassis Cardiac Surgery Center (Hospital 2), were informed about the study and participated after signing an informed consent.

A brief questionnaire was administered to HCWs with information about age, gender, education, position within hospital, BMI, history of risk factors for severe COVID-19 (RFS-CoV), previous COVID-19 (Pr-CoV) and history of self-reported adverse reactions after vaccination (VSE). The VSE were grouped according to the major symptom as local, allergic reactions, fever, fatigue and systematic. Combinations of VSE were counted in each one of the individuals’ groups.

### 2.2. Natural Infection Group

A group of 315 patients with natural SARS-CoV-2 infection diagnosed with RT-PCR testing was included in the study. Participants provided informed consent. Age, gender, diagnostic tests, symptoms, hospitalization, disease severity [19] and admission in intensive care unit (ICU) were recorded. The present analysis includes in total 180 patients, 157 hospitalized and 23 non-hospitalized, 171 symptomatic and 9 asymptomatic individuals, 155 patients with available time from symptom onset (PSO) and 163 with estimation of severity.

The study was approved by the IRB committees of Laiko General Hospital (Athens, Greece) and Onasis Cardiac Surgery Center (Kallithea, Greece).

### 2.3. Serological Tests

Serum samples collected after venipuncture were tested for SARS-CoV-2 IgG binding antibodies to nucleocapsid protein (anti-N) and anti-SARS-CoV-2 receptor binding domain (RBD) spike protein IgG (anti-RBD).

The first assay is a qualitative one with an index [sample/calibrator (s/c)] cutoff of 1.4. Samples with an index ≥ 1.4 are considered positive and <1.4 negative. The clinical sensitivity of the anti-N assay in samples collected ≥15 days after onset of symptoms is 100% (95% CI 95.9–100%) and the clinical specificity 99.63% (95% CI 99.05–99.90%), according to the manufacturer [20].

The second assay (Abbott SARS-CoV-2 IgG II Quant) or anti-RBD was used to quantify IgG antibodies against the receptor binding domain (RBD) of the S1 subunit of the spike protein. The linear range is between 21 and 40,000 AU/mL. The lower limit of detection was 6.8 AU/mL and the reportable interval 6.8–80,000 AU/mL. The clinical sensitivity was 98.81% (95% CI 93.56–99.94%) in samples collected ≥ 15 days after the positive PCR and the clinical specificity 99.55% (95% CI 99.15–99.76%), at a cutoff value 50 AU/mL [20].

Both assays are based on chemiluminescent microparticle immune assay (CLIA) [20].

The correlation coefficient in weighted linear regression of WHO standard with the Abbott anti-RBD is 0.999, and transformation of Abbott anti-RBD AU/mL to WHO BAU/mL is feasible using the equation BAU/mL = 0.142 × AU/mL [20].

### 2.4. Statistical Analysis

Median values, 25th and 75th percentiles, were used to describe anti-RBD levels. We compared levels between vaccinated health care workers and individuals with a prior SARS-CoV-2 infection diagnosis or other groups using the non-parametric Mann–Whitney U test or Kruskal–Wallis test. Multiple linear regression was used to identify factors associated with anti-RBD levels. Ratios of the median anti-RBD levels among vaccinated people versus the median levels among persons with natural immunity (asymptomatic, mild symptoms and moderate/severe symptoms) were also calculated.

We conducted all statistical analyses using STATA 13.1. Figures were created in R (v 4.1.0).

## 3. Results

### 3.1. Vaccinated HCWs

Eight hundred seventy-one HCWs participated in the study. Their sociodemographic characteristics are shown in Table 1.

The prevalence of the SARS-CoV-2 anti-N IgG was 3.7% (32 out of 871) (95% CI 2.5–5.2%) and anti-RBD IgG was 99.7% (868 out of 871) (95% CI 99.0–99.3%). The concentrations of anti-RBD ranged from <6.8 up to higher than 80.000 AU/mL. The 2.5th, 50th and 97.5th percentiles of anti-RBD were 1680, 15,877 and 55,309 AU/mL, respectively (Figure S1A,B).

The median (IQR) anti-RBD levels by age, gender, country of birth, BMI, risk factors for severe COVID-19, side effects of vaccination and previous SARS-CoV-2 infection are shown in Table 2. Gender, age, previous SARS-CoV-2 infection, side effects of vaccination and risk factors for COVID-19 showed statistically significant association with concentrations of anti-RBD. However, in a multivariable linear regression analysis only gender, age, side effects of vaccination and previous SARS-CoV-2 infection showed statistically significant associations with concentrations of anti-RBD (Table 2). More specifically, females had, on average, a 2823 (95% CI 859–4787) AU/mL concentration higher than male HCWs (*p* = 0.05). Participants aged 55–70 yrs had, on average, a 7651 (95% CI 4479–10,823) AU/mL lower concentration than HCWs 18–34 yrs (*p* < 0.001).

HCWs reporting VSE had a concentration of 5024 (95% CI 3122–6926) AU/mL higher than those not reporting side effects (*p* < 0.001).

Among HCWs, 496 individuals reported 988 VSE, ranging from 1 to 6 per HCW. HCWs reporting fever, fatigue, local or other systematic reactions had statistically significantly higher concentrations of anti-RBD. Fever was associated with 2.3 times higher levels of anti-RBD compared with no VSE (Table 2).

**Figure 1:**
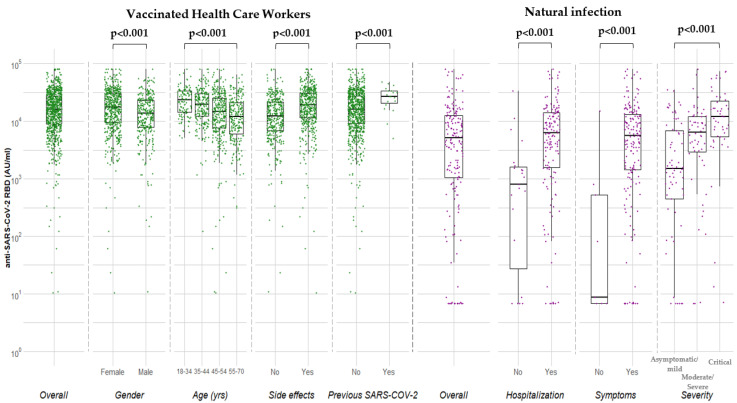
Median concentrations of anti-SARS-CoV-2 RBD (AU/mL) in vaccinated health care workers 7–15 days after the 2nd dose of BNT162b2 and individuals with natural infection.

HCWs with previous SARS-CoV-2 had higher levels by 9971 (95% 5158–14,783) AU/mL compared to COVID-19 naïve individuals (*p* < 0.001). Figure 1 depicts the median levels of anti-RBD overall and by gender, age, side effects and previous SARS-CoV2 in vaccinated HCWs.

Time from 2nd dose: The median levels of anti-RBD were calculated according to the time from the 2nd dose. The maximum levels were reached 11 days after the 2nd dose while a sharp reduction was observed 15–17 days after the 2nd dose (Kruskal–Wallis, *p* = 0.007) (Table S1). Multivariable analysis showed that reduction was independent of age, gender, side effects of vaccination and previous SARS-CoV-2 infection (data not shown).

### 3.2. Natural Infection

The early convalescent samples post-symptoms onset (PSO) ≥15–59 days of symptomatic (*n* = 155), asymptomatic individuals (*n* = 9), hospitalized (*n* = 157) and non-hospitalized (*n* = 23) individuals were included in this analysis. The sociodemographic and clinical characteristics are shown in Table 3.

The prevalence (95% CI) of anti-N and anti-RBD was 88.3% (82.7–92.6%) and 90.6% (85.3–94.4%), respectively.

The median (IQR) anti-RBD levels by age, gender, symptomatic/asymptomatic, severity of clinical disease and time POS are shown in Table 4. The median (IQR) anti-RBD concentrations were 9 (<6.8–520) and 5547 (1415–13,325) AU/mL in asymptomatic and symptomatic individuals, respectively (*p* < 0.001), 6271 (1583–14,121) and 808 (9-1668) hospitalized and non-hospitalized, respectively.

The median (IQR) anti-RBD levels were highly associated with increased severity: mild, 1634 (751–7868); moderate, 6082 (2433–12,224); severe, 6638 (3053–13,837); and critical, 11,975 (5138–23,351) AU/mL (*p* < 0.001 for between group comparisons).

**Figure 2:**
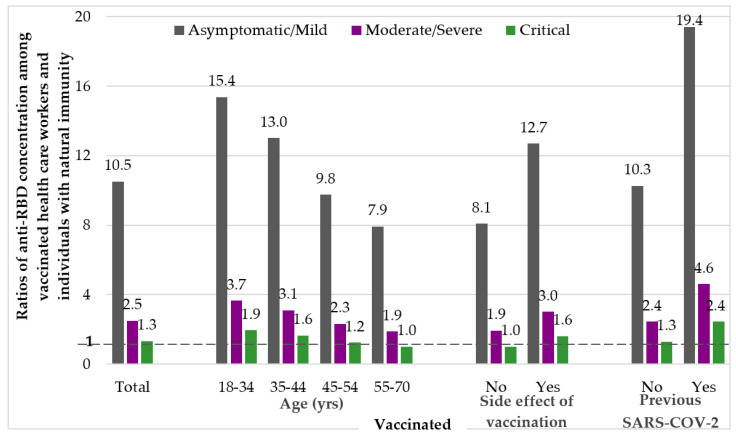
Ratio of median concentrations of anti-SARS-CoV-2 RBD in vaccinated groups versus naturally infected individuals with asymptomatic/mild, moderate/severe and critical infection.

Hospitalized individuals had 7.8-fold higher median anti-RBD levels than those who were not hospitalized. Specifically, patients with moderate, severe, and critical disease had a 4.0-, 4.4-, 7.9-fold higher anti-RBD level than those with asymptomatic/mild infection, respectively (Figure 2).

### 3.3. Comparison of Anti-RBD Levels in Vaccinated HCWs and in Individuals with Natural Infection

Figure 1 depicts the median anti-RBD levels in naturally infected individuals overall and according to hospitalization, symptoms and severity. The ratio of median anti-RBD levels in vaccinated after the 2nd dose versus the median levels of those with natural infection in the early convalescent period 15–59 days POS is shown in Figure 2. Anti-RBD concentrations of natural infection were used as denominators (asymptomatic/mild, moderate/severe and critical infection).

We observed several-fold differences in the anti-RBD ratio for each vaccinated group, e.g., across different age groups, i.e., 18–34, 35–44, 45–54, 55–70 years old, and the ratio of median anti-RBD levels for vaccinated over natural infection was 1.9–15.4, 1.6–13.0, 1.2–9.8 and 1.0–7.9, respectively. In the group with VSE the ratio was 1.6–12.7 and in the group with Pr-CoV 2.4–19.4 (Figure 2). For the whole group of vaccinated individuals, the ratio was 1.3, 2.5, 10.5 based on critical, moderate/severe and asymptomatic/mild patients with natural infection, respectively.

## 4. Discussion

Several lines of evidence suggest that neutralizing antibodies are correlates of protection (CoP) against SARS-CoV-2 infection.

Studies on macaques infected by SARS-CoV-2 demonstrated the pivotal role of NA and S specific CD4+ and CD8+ T-Cell responses to provide near complete protection in rechallenge experiments [21].

Studies in non-human primates vaccinated by SARS-CoV-2 vaccines demonstrated NA threshold for complete protection [22,23].

A study of natural infection outbreak in a fishery vessel where, of the 117 individuals who were seronegative to NA and binding antibodies prior to departure, 104 were (88.9%) infected by RT-PCR while, of the three crew members with presence of NA, anti-RBD and antibodies to full-length spike, none were infected [24].

A prospective study in 3.168 marine recruits revealed aggregate infection of 48% during a 6-week training period. Among 189 anti-spike and anti-RBD-positive cases, 19 (10%) were infected by RT-PCR during training. Lower levels of neutralizing and binding antibodies were associated with a higher incidence of infection [25].

A higher AZD1222 vaccine efficacy was demonstrated with higher levels of NA and anti-spike IgG in a vaccination trial [26]. Moreover, anti-RBD and anti-Spike binding assays were equivalent to NA in predicting vaccine efficacy for predicting symptomatic infection [26]. An excellent correlation of anti-RBD and NA was observed in vaccination trials [27,28,29,30] or other studies [6,7,9].

In a recent study including non-human primates, purified spike antibodies elicited by mRNA-1273 vaccine were associated with a dose-dependent protection in a highly pathogenic hamster model [31].

A remarkable finding of mRNA vaccines immunogenicity studies is that levels of anti-RBD and neutralizing antibodies titers do not change after the 2nd dose in individuals previously infected, suggesting that the 2nd dose of BNT122b2 or other vaccines may not be necessary in previously infected immunocompetent individuals [30,31,32]. In our study, we tested vaccinated HCWs by anti-N and anti-RBD 1–2 weeks after the 2nd dose. Thirty-two HCWs were found to be positive for anti-N, which is a prevalence of 3.2% (32/871) (95% CI 2.5–5.2%). Only 19/32 (59.4%) had a history of previous COVID-19 diagnosis by RT-PCR, suggesting that anti-N is a useful test to assess previous COVID-19 infection during vaccination. Both groups of anti-N-positive subjects had significantly higher anti-RBD levels compared with HCWs without previous SARS-CoV-2 infection.

Studies of BNT162b2 immunogenicity after the 1st and 2nd dose found various associations with age, gender, obesity, vaccination side effects and previous COVID-19. However, confounding effects were not controlled by multivariable analysis [33,34,35]. In our study, anti-RBD levels significantly decreased with older age, male gender, presence of risk-factors for COVID-19 and increased with side-effects and previous SARS-CoV-2 infection. After multivariable analysis, a significant association with age, gender, side-effects of vaccination and previous COVID-19 remained.

We further studied a large group of individuals with natural infection including 175 symptomatic and 26 asymptomatic people diagnosed with RT-PCR and available demographic and clinical information. Sera were collected 15–59 days POS in symptomatic individuals. To our knowledge, this is the largest and more comprehensive natural infection panel included in SARS-CoV-2 vaccine immunogenicity studies. The immunogenicity difference of moderate/severe and asymptomatic infection exceeds 1 log10 and it may bias immunogenicity comparisons in vaccine studies. By using asymptomatic infection as the baseline group, the anti-RBD concentration was several-fold higher in vaccinated individuals compared with natural infection. In contrast, by using moderate/severe infection, the vaccinated individuals had slightly elevated concentrations of anti-RBD compared to patients with natural infection.

In a phase 1 study assessing the immunogenicity of a candidate mRNA vaccine, the ratio of anti-RBD and NA in vaccinated vs. hospitalized or non-hospitalized patients with natural infection was 0.15 (anti-RBD, hospitalized), 0.76 (anti-RBD, non-hospitalized), 0.18 (NA, hospitalized) and 1.0 (NA, non-hospitalized), respectively [36]. Indeed, vaccine manufacturers recently released data which revealed that vaccine efficacy was 47% [37], verifying that a suboptimal immunogenicity was associated with lower efficacy of the candidate vaccine. These data strongly support that vaccine efficacy can be predicted by comparisons of immunogenicity in few vaccinated individuals with serological panels from natural infection, including patient sera from all COVID-19 stages.

Overall, these data document the high immunogenicity of BNT162b2 vaccine in comparison with natural infection. Both mRNA BNT1162b2 and mRNA 1273 vaccines are highly immunogenic although emergence of SARS-CoV-2 variants deserve careful consideration.

Novel SARS-CoV-2 variants harboring mutations can either enhance transmissibility or reduce neutralization activity of vaccine sera. Variants of concern and include the B.1.1.7, B.1.351, P.1 and B.1.617.2 documented first in the UK, South Africa, Brazil and India, respectively. The effectiveness of the BNT162b2 vaccine was assessed under real-world conditions in Qatar, where B.1.1.7 and B.1.351 variants were co-circulating [38]. Specifically, the effectiveness of the vaccine against the B.1.1.7 and B.1.351 documented infections was 89.5% (95% CI, 85.9 to 92.3) and 75.0% (95% CI, 70.5 to 78.9), respectively, suggesting that the mRNA vaccine can protect against immune escape variants of concerns, such as the B.1.351. Recent data suggest effectiveness of BNT162b2 against B.1.167.2 variant [39]. Overall, despite the BNT162b2 effectiveness in the presence of variants, it is wise to maintain high immunogenicity levels to secure adequate vaccine efficacy [40].

The present analysis assumes according to findings from previous reports that NA and anti-RBD are correlates of protection against SARS-CoV-2 [5,21,22,23,24,25,26,31]. Although the mechanism of protection is complex including several aspects of humoral and cell-mediated immunity, the role of neutralizing antibodies seems profound. The use of multiple NA assays, many of them requiring increased biosafety laboratories is a barrier for large clinical studies. The use of anti-RBD or other antibodies against spike protein may accelerate the study of pathogenesis of SARS-CoV-2 and provide useful tools for assessing vaccine efficacy and their effectiveness over time. Expensive laboratory testing (functional NAs, cellular immunity testing) may be limited in phase 1/2 studies, and vaccine safety evaluation should be assessed in large observational studies without conducting expensive and time-consuming randomized phase 3 clinical trials [41].

This study has some strengths including the large number of vaccinated individuals evaluated, the use of a large natural infection panel and the use of a validated, commercially available assay for anti-RBD testing. The major limitation is the lack of cell-mediated immunity tests to capture aspects of T and B cell immunity. The study is also limited in assessing anti-RBD as correlate of protection without further assessment of other antibody targets or functional aspects of humoral immune response.

## 5. Conclusions

Prospective evaluation of vaccine immunogenicity in large, vaccinated cohorts is underway and the comparison, with prospective data from natural infection potentially clarifying important questions of COVID-19 pathogenesis and vaccine effectiveness over time.

## Figures and Tables

**Table 1:** Sociodemographic and clinical characteristics of health care workers participating in immunogenicity studies.

	N (%)
**Total**	871 (100.0)
**Gender**	
Male	318 (36.5)
Female	553 (63.5)
**Age (y)**	
**Mean (SD)**	47.8 (10.3)
18–34	113 (13.0)
35–44	215 (24.7)
45–54	315 (36.2)
55–70	228 (26.2)
**Country of Birth**	
Greece	804 (92.3)
Other	67 (7.7)
**Hospital**	
1	514 (59.0)
2	357 (41.0)
**Risk Factors for Severe COVID-19**	
Yes	134 (15.7)
No	721 (84.3)
**Job title**	
HCWs involved with the patient care	709 (81.4)
HCWs not involved with the patient care	162 (18.6)
**History of SARS-CoV-2 infection**	
Yes	32 (3.7)
No	839 (96.3)

**Table 2:** Median (25th, 75th) concentration of anti-SARS-CoV-2 IgG-II antibodies after the second dose of BNT162b2 vaccine and coefficients (β) along with 95% Confidence Intervals from multiple linear regression.

Covariate	N (%)	Median (25th, 75th) (AU/mL)	*p*	β (95% CI)	*p*
**Overall**	871 (100.0)	15,877 (8854–27,355)		–	
**Gender**			<0.001		
Male	318 (36.5)	13,661 (7780–23,245)		Ref.	
Female	553 (63.5)	17,711 (9678–29,726)		2823 (859–4787)	0.005
**Age (y)**			<0.001		
18–34	113 (13.0)	23,248 (14,447–33,403)		Ref.	
35–44	215 (24.7)	19,669 (12,210–29,683)		−2466 (−5583–651)	0.121
45–54	315 (36.2)	14,748 (7636–25,363)		−6228 (−9203–−3254)	<0.001
55–70	228 (26.2)	11,977 (5993–21,101)		−7651 (−10,823–−4479)	<0.001
**Country of birth**			0.524		
Greece	804 (92.3)	15,612 (8785–26,994)		–	
Other	67 (7.7)	17,293 (9569–28,664)		–	
**Risk factors for COVID-19**			0.066		
No	721 (84.3)	16,289 (9348–27,506)		Ref.	
Yes	134 (15.7)	13,374 (7422–25,044)		−246 (−2800–2309)	0.850
**BMI (kg/m^2^)**			0.125		
Under/Normal weight: <25	383 (44.0)	16,692 (9597–29,375)		–	
Overweight: 25–30	323 (37.1)	14,823 (7931–24,804)		–	
Obesity: ≥30	165 (18.9)	15,525 (8326–25,606)		–	
**Side effects of vaccination**					
No	375 (43.1)	12,210 (6848–21,298)	Ref.	Ref.	
Yes	496 (56.9)	19,196 (11,334–30,841)	<0.001 ^a^	5024 (3122–6926)	<0.001
Fever	128 (25.81)	28,687 (16,207–39,100)	<0.0001 ^b^		
Fatigue	287 (57.9)	19,616 (12,137–31,255)	<0.0001 ^c^		
Allergic reactions	14 (2.8)	16,163 (9947–27,997)	0.2792 ^d^		
Local	198 (39.9)	18,425 (10,086–30,300)	<0.0001 ^e^		
Other systematic	248 (50.0)	21,692 (12,229–33,381)	<0.0001 ^f^		
**Previous SARS-COV-2**			<0.001		
No	839 (96.3)	15,520 (8710–26,480)		Ref.	
Yes	32 (3.7)	29,324 (17,751–41,821) ^g^		9971 (5158–14,783)	<0.001
PCR Positive (+)	19 (59.4)	26,986 (19,212–33,866) ^h^			
No history of PCR testing	13 (40.6)	33,950 (9947–49,915) ^i^			

*p*-values for pairwise comparisons to the reference value are presented: ^a^ *p* < 0.001, ^b^ *p* < 0.001, ^c^ *p* < 0.000, ^d^ *p* = 2792, ^e^ *p* < 0.000, ^f^ *p* < 0.000, ^g^ *p* < 0.00, ^h^ *p* = 0.0017, ^i^ *p* = 0.0167.

**Table 3:** Sociodemographic and clinical characteristics of individuals with COVID-19 infection participating in immunogenicity studies.

Variable	N (%)
**Total**	180 (100.0)
**Gender**	
Male	126 (70.0)
Female	54 (30.0)
**Age (y)**	
**Mean (SD)**	59.6 (16.7)
≤54	63 (36.4)
55–64	42 (24.3)
≥65	68 (39.3)
**Hospitalization**	
No	23 (12.8)
Yes	157 (87.2)
**Symptoms**	
Symptomatic	171 (95.0)
Asymptomatic	9 (5.0)
**Severity of Symptoms**	
Mild	60 (36.8)
Moderate	39 (23.9)
Severe	17 (10.4)
Critical	47 (28.8)

**Table 4:** Median (25th, 75th) concentration of anti-SARS-CoV-2 IgG-II antibodies in symptomatic SARS-CoV-2, 15–59 days after infection and asymptomatic individuals.

Covariate	N (%)	Median (25th–75th) (AU/mL)	*p*
**Overall**	180 (100.0)	5088 (1050–12,620)	
**Gender**			0.2691
Male	126 (70.0)	5632 (1273–13,837)	
Female	54 (30.0)	3743 (883–11,187)	
**Age (y) ^a^**			0.0603
18–44	33 (19.1)	1297 (296–7519)	
45–54	30 (17.3)	6577 (1601–16,623)	
55–64	42 (24.3)	6868 (2433–11,975)	
65+	68 (39.3)	5814 (1553–13,110)	
**Hospitalization**			<0.001
No	23 (12.8)	808 (9–1668)	
Yes	157 (87.2)	6271 (1583–14,121)	
**Asymptomatic**			0.0005
No	171 (95.0)	5547 (1415–13,325)	
Yes	9 (5.0)	9 (<6.8–520)	
**Severity ^b^**			0.0001
Mild	60 (36.8)	1634 (751–7868)	
Moderate	39 (23.9)	6082 (2433–9579)	
Severe	17 (10.4)	6638 (3053–13,837)	
Critical	47 (28.8)	11,975 (5318–23,351)	
**Time from symptoms onset (days) ^c^**			0.8887
15–29	82 (52.9)	6980 (1060–14,968)	
30–44	48 (31.0)	6122 (2169–13,925)	
45–59	25 (16.1)	5452 (3306–9579)	

^a^ Seven missing values, ^b^ Eight missing values, ^c^ Sixteen missing values. When grouped differently: Asymptomatic/mild: 1512 (452–6862) AU/mL and Moderate/Severe: 6360 (2895–12,137) AU/mL.

## Data Availability

All relevant data are available at the Pergamos Institutional Repository of the National and Kapodistrian University of Athens, Greece. Link available upon review of manuscript.

## Supplementary Materials

The following are available online at https://www.mdpi.com/article/10.3390/vaccines9091017/s1, Supplementary Figure S1A: Cumulative distribution of anti-SARS-CoV-2 RBD AU/mL in vaccinated health care workers 5–17 days after the 2nd dose of BNT162b2, Supplementary Figure S1B: Frequency distribution of anti-SARS-CoV-2 RBD AU/mL in vaccinated health care workers 5–17 days after the 2nd dose of BNT162b2, Supplementary Table S1: Median (25–75th) levels of anti-SARS-CoV-2 RBD IgG by time (days) from 2nd dose of BNT162b2 vaccine.
