# Supplementary material for: Comparative Immunogenicity of BNT162b2 mRNA Vaccine with Natural SARS-CoV-2 Infection
Source: Vaccines (Basel). 2021 Sep 13;9(9):1017. doi: 10.3390/vaccines9091017 (PMC8471735; doi:10.3390/vaccines9091017)
Supplement: Supplementary file 1 [file vaccines-09-01017-s001.zip › vaccines-1359690-supplementary.pdf]

Supplementary Table S1. Median (25th-75th) levels of anti-SARS-CoV-2 RBD IgG by time (days) from 2nd dose of BNT162b2 vaccine

	N (%)	Median (25th-75th) (AU/ml)	P[a]
**Days from second dose**			0.007
5-7	131 (15.0)	15,107 (6,736 - 26,119)	
8	142 (16.3)	16,503 (9,678 - 29,683)	
9	128 (14.7)	17,514 (9,867 - 27,522)	
10	107 (12.3)	17,081 (9,531 - 28,318)	
11	56 (6.4)	19,830 (13,525 - 30,796)	
12	88 (10.1)	15,229 (10,274 - 29,429)	
13	80 (9.2)	15,706 (8,557 - 23,497)	
14	92 (10.6)	12,462 (7,519 - 27,151)	
15-17	47 (5.4)	10,330 (5,529 - 18,880)	

Abbreviations: 25th–75th, 25th and 75th percentiles; AU/ml, arbitrary units per milliliter

[a]Nonparametric Kruskal-Wallis test.

Figure Legends

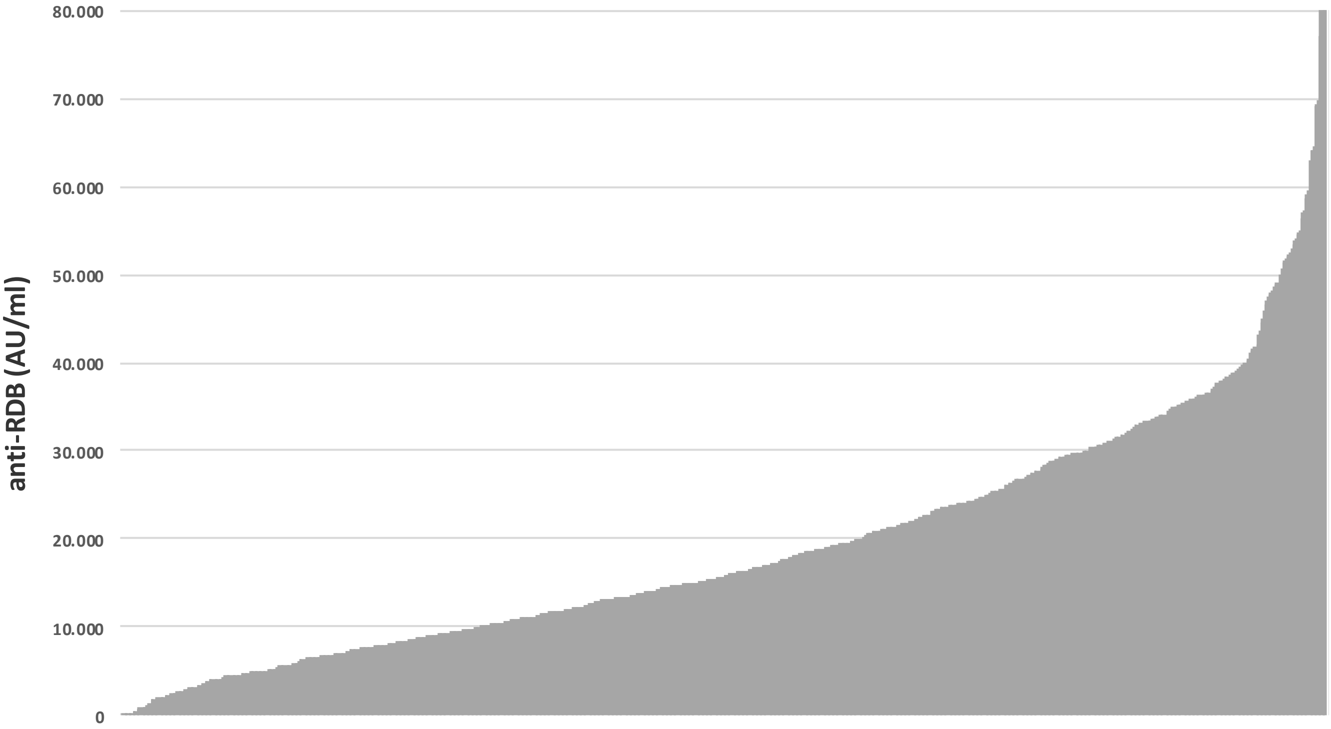

(A)

Supplementary Figure S1A. Cumulative distribution of anti-SARS-CoV-2 RBD AU/ml in vaccinated health care workers 5-17 days after the 2nd dose of BNT162b2.

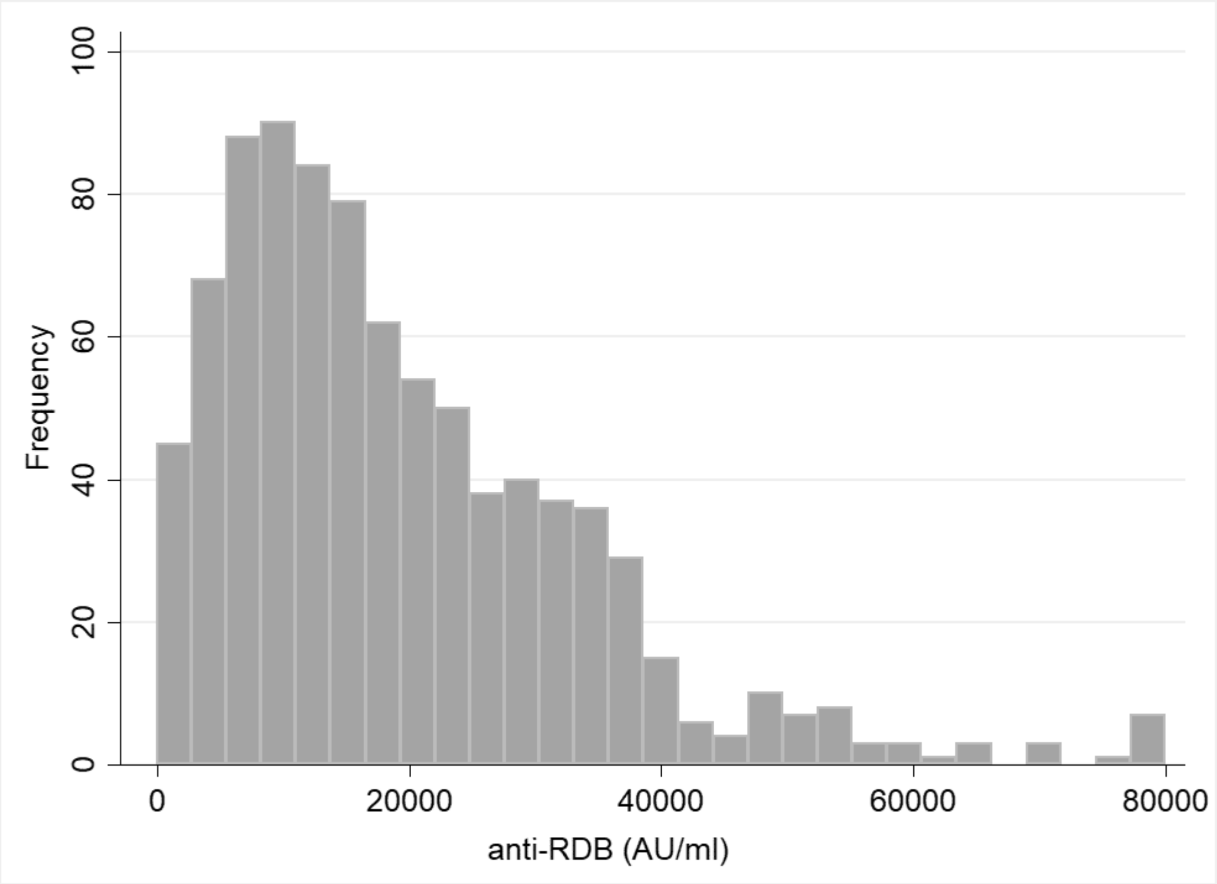

(B)

Supplementary Figure S1B. Frequency distribution of anti-SARS-CoV-2 RBD AU/ml in vaccinated health care workers 5-17 days after the 2nd dose of BNT162b2.
